# HIV, syphilis and behavioral risk factors among men who have sex with men in a drug-using area of southwestern China: Results of 3 cross-sectional surveys from 2013 to 2015

**DOI:** 10.1097/MD.0000000000010404

**Published:** 2018-04-20

**Authors:** Lan Guanghua, Chen Yi, Tang Shuai, Shen Zhiyong, Tang Zhenzhu, Ruan Yuhua, Mohammed Adnan Yousuf, Fan Wensheng

**Affiliations:** a Guangxi Center for Disease Control and Prevention, Nanning; b State Key Laboratory for Infectious Disease Prevention and Control (SKLID), Chinese Center for Disease Control and Prevention (China CDC), Beijing; c Collaborative Innovation Center for Diagnosis and Treatment of Infectious Diseases, Hangzhou, China; d Department of Public Health, Western Kentucky University, Bowling Green, KY; e Department of Health Services Administration, Florida International University, Miami, FL.

**Keywords:** behavioral risk factors, China, HIV, men who have sex with men, syphilis

## Abstract

To assess human immunodeficiency virus (HIV), syphilis, and behavioral risk factors among men who have sex with men (MSM) in southwestern China, where HIV started as a drug-driven epidemic, and shifted to mainly heterosexual transmission.

These cross-sectional studies were conducted yearly in 2013, 2014, and 2015 in Guangxi, China. A total of 1,996, 1,965, and 1,697 participants were recruited in 2013, 2014, and 2015, respectively. The data included demographic and sexual behavioral variables. Other variables included individuals who used illegal drugs, and who received HIV counseling, testing, and free condoms, and peer education. Participants were tested for HIV, syphilis, and hepatitis C virus (HCV) with whole blood specimens. Questionnaires and laboratory testing data were double entered, and validated with EpiData software. The data were then transferred into SPSS software (SPSS Inc, Chicago, IL) and Chi-square test performed.

The prevalence of HIV was 6.6% in 2013, 8.4% in 2014, and 11.2% in 2015. The prevalence of syphilis was 9.3% in 2013, 9.8% in 2014, and 6.1% in 2015. And HCV prevalence was 0.5% in 2013 and remained stable at 0.4% in 2014, and 2015. HIV infection, and associated factors among MSM in these 3 annual cross-sectional survey showed that HIV-infected MSM were significantly, more likely, to perform unprotected anal intercourse with any commercial male partners in the past 6 months (adjusted odds ratio [AOR] = 1.81, 95% CI: 1.50–2.20), had sex with any female partners in the past 6 months (AOR = 1.31, 95% CI: 1.01–1.71), used drugs in the past (AOR = 2.73, 95% CI: 1.30–5.71), and are syphilis infected (AOR = 3.53, 95% CI: 2.77–4.49).

There is an urgent need for intervention strategies like condom distribution, HIV counseling, free testing, and education regarding safe sex, HIV, and other sex-related diseases in Guangxi to curb, and prevent HIV among MSM.

**Article summary**

**Strengths and limitations of this study:**

- Among all provinces, and municipalities in China, the Guangxi Zhuang Autonomous Region contains among the highest number of reported HIV cases.
- This is the first study to assess changes of HIV, syphilis infections, and behavioral risk factors among MSM in southwestern China, where HIV started as a drug-driven epidemic, and shifted to mainly heterosexual transmission.
- Currently, of the total number of reported HIV cases in China, about 12% were found in Guangxi.
- Ninty three percent of reported HIV cases in Guangxi were infected through heterosexual intercourse in the year 2013.
- The proportion of reported HIV/AIDS through homosexual intercourse was low (2.3%) in the year 2013 in Guangxi. This low report rate could lead to fewer HIV preventative measures being carried out in the future in Guangxi MSM.
- The prevalence of HIV was 6.6% in the year 2013 and gradually rose to an alarming rate of 11.2% in the year 2015.

## 1 Background

Due to China adopting an open-door policy in the late 1980s, drug abuse first emerged as a problem in China.^[1,2]^ The majority of illicit drugs in China were brought from Vietnam into Guangxi, or Myanmar into Yunnan, and then into other sites such as Sichuan, and Xinjiang.^[3]^ Thereafter, a small proportion of heroin was trafficked from the “Golden Crescent” to Xinjiang, and then into other neighboring provinces.^[4,5]^ According to a report by the National Narcotic Control Commission (NNCC), 88% of all drug users used heroin in 2002.^[6]^

The first human immunodeficiency virus (HIV) infection among injection drug users (IDU) in China was found in the southwestern province of Yunnan in 1989.^[7,8]^ It then increased transmission rates of HIV among IDUs residing along the major drug-trafficking roads to Guangxi, Sichuan, Xinjiang, and other provinces.^[3,9]^ By the end of 2002, HIV infections among IDUs had been reported in all 31 mainland provinces, and injection drug use contributed to 70.9% of the total reported HIV/ acquired immune deficiency syndrome (AIDS) cases in the China.^[10]^ During this period, commercial sex activities flourished in China due to China adopting a free market economy, and an open door policy in 1978.^[11]^ The proportion of reported HIV/AIDS through heterosexual intercourse was increased from 22.8% in 2007 to 69% in 2013,^[12]^ while HIV/AIDS in IDU's reduced from 29% in 2007 to 6% in 2014.^[13]^ Recent studies show that approximately, 17.4% of new HIV infections are spread through homosexual transmission.^[14]^ Studies in China show that between 2 and 5% of sexually active Chinese men have sex with other men, which accounts a total between 2 and 8 million Chinese male population.^[15]^ The prevalence of HIV among men who have sex with men (MSM) is ascending at a high rate. The proportion of reported HIV/AIDS cases among MSM increased from 1.7% in 2009 to 21.0% in the year 2013.^[14]^ According to Chow et al almost 5.3% of MSM are infected with HIV, which surpasses the HIV rate 90 times when compared to general Chinese population.^[15]^ China followed similar trends in the HIV epidemic as other countries, beginning as a drug-driven epidemic, and shifting to mainly sexual transmission.^[16]^

The Guangxi Zhuang Autonomous Region is located on the southern coast of China. It borders Vietnam to the southwest, Guizhou to the northwest, Yunnan to the west, Hunan to the northeast, and Guangdong to the east. Due to its location along the major drug trafficking route linking Guangxi with Yunnan, and Vietnam, the HIV transmission is fueled mainly by injection drug use.^[17,18]^ Since the first HIV infection among local IDUs was discovered in the year 1996 in Guangxi, HIV infections through injecting drugs accounted for 69% of the total reported cases of HIV in Guangxi in the year 2003.^[19]^ With passing years heterosexual became the prominent route for HIV transmission in Guangxi. In the year 2007 there were 42.8% reported cases, which increased to 78.2% in the year 2010, and 90% in the year 2012.^[20,21]^ Among all provinces, and municipalities in China, the Guangxi Zhuang Autonomous Region has possessed the second highest number of reported HIV cases.^[20,22]^ This is the first study to assess HIV, syphilis, and behavioral risk factors among MSM in southwestern China, where HIV started as a drug-driven epidemic, and shifted to mainly heterosexual transmission.

## 2 Methods

### 2.1 Study design and participants

These cross-sectional studies were conducted yearly in 2013, 2014, and 2015 in 8 of 14 leading cities in Guangxi, China: Guigang, Guilin, Hechi, Hezhou, Liuzhou, Nanning, Wuzhou, and Yulin. Participants for the study were recruited in 3 ways. The first method employed was advertisements through local gay websites, and QQ chat room groups. For the second process, peer recruiters were trained to reach out to the homosexual community in locations such as bars, parks, bathhouses, and clubs frequented by MSM. Peer recruiters distributed flyers with study-related information. The third method constituted study participants being encouraged to disseminate study information, and refer their peers to participate in the study. Eligibility criteria required participants to be at least 18 years of age, to self-report same-gender sex in the past 6 months, to be local residents, and to provide written informed consent. Written informed consent was obtained from all study participants before the survey. Those who met the screening criteria then completed a data collection interview, received an HIV pretest, and counseling, and had blood drawn to test for HIV, syphilis, and hepatitis C virus (HCV) antibodies. Participants were also given HIV post-test counseling when they subsequently, returned for their HIV test results. Later, the affected individuals were referred to the local centers for treatment, and were provided care. The study protocol, and informed consent form were approved by the Institutional Review Board (IRB) of the Guangxi Center for Disease Control and Prevention (CDC).

### 2.2 Data collection

The health staff from the local CDC received the training for protocol, ethical, interview, counseling, and data collection. Structured questionnaire-based interviews were carried out face-to-face by the trained health professionals on a one-on-one basis in a private room. This questionnaire was adopted from the Chinese national sentinel surveillance^[23–25]^ Each participant was assigned a unique, and confidential identification number for the questionnaire, and blood specimens. Demographic variables collected were age, ethnicity, marital status, education, residence, city, and Internet recruitment status. Behavioral variables include participants having anal intercourse with any male partners, having anal intercourse with any commercial male partners, having sex with any female partners, using illegal drugs, receiving peer education, and receiving HIV counseling, testing, and condoms.

### 2.3 Laboratory tests

Of all the 1,996 participants in the year 2013, 1,965 participants in the year 2014, and 1,697 participants in the year 2015 blood samples were collected for serologic testing for HIV, syphilis, and HCV. Blood samples were tested for HIV antibodies with an enzyme-linked immuno sorbent assay (ELISA) (Beijing Wantai Biological Medicine Company, China). Western blot tests were conducted to confirm the positive HIV infection (HIV Blot 2.2 WB, Genelabs Diagnostics, Singapore). For syphilis infections, all blood specimens were tested with ELISA (Beijing Wantai Biological Production Company, China), and the rapid plasma regain (RPR) test (Shanghai Rongsheng, China). Specimens with reactivity on both tests were confirmed to have current syphilis. HCV was tested with an ELISA (Beijing Wantai Biological Production Company, China).

### 2.4 Data analysis

Questionnaires and laboratory testing data were double entered and validated with EpiData software (EpiData 3.0 for Windows; The EpiData Association Odense, Denmark). The data were then transferred into SPSS software (version 17.0; SPSS Inc, Chicago, IL). To assess crude differences among the years 2013, 2014, and 2015 cross-sectional studies, Chi-square tests for trends were used for categorical variables. A stepwise multivariate logistic regression model was constructed to select the variables that were independently, associated with HIV infection. *P* value <.05 was considered statistically, significant, and all tests were 2-sided.

## 3 Results

A total of 1996, 1965, and 1697 participants were recruited during the years 2013, 2014, and 2015, respectively. Of the 5698 participants recruited, and screened between the years 2013 and 2015, 40 were excluded from the study, as 6 participants were younger than 18 years of age, 7 participants said that they did not have sex in past 6 months, and 27 participants were not willing to participate in the survey. The survey response rate was 99.3%. Participant's sociodemographic characteristics are presented in Table 1, showing the statistical difference, which is a general multi-group Chi-square test. Most of the variables are different across all 3 years with no clear trend. In all the surveys, a majority of participants were greater than, or equal to 25 years of age, with more than two-thirds belonging to Han ethnic group. The following characteristics show a significant relationship. A majority of participants were single, divorced, or widower (*P* < .001). The percentage of participants recruited from the Internet rose from 55.9% in the year 2013 to 73.0% in the year 2015 (*P* < .001). The majority of participants resided in Nanning, and a minimum of participants resided in Yulin, between the years 2013 and 2015.

**Table 1:**
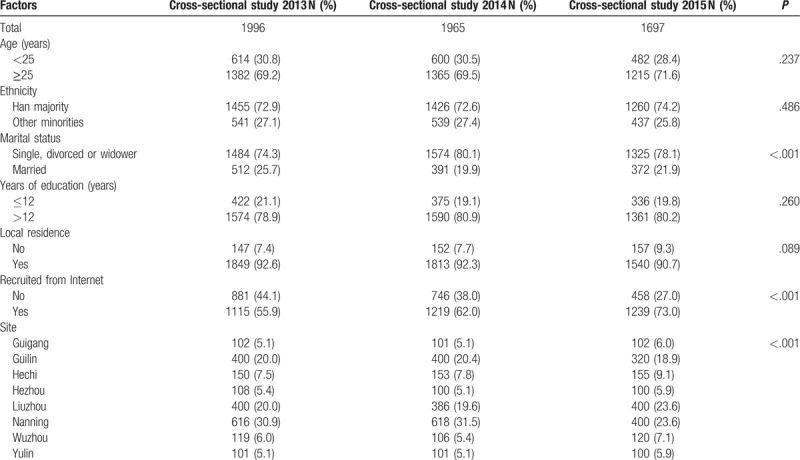
Socio-demographic characteristics of participants in cross-sectional study 2013, 2014 and 2015.

Table 2 shows changes of participants’ behavioral characteristics, and HIV, and Syphilis status of participants over time between the years 2013 and 2015. Proportion of MSM who had anal intercourses with any male partners in past 6 months increased from 77.5% in the year 2013 to 83.7% in the year 2014, and decreased to 82.3% in the year 2015 (*P* < .001); the proportion who had anal intercourses with any commercial male partners in the past 6 months decreased from 7.9% in the year 2013 to 3.3% in the year 2014, and raised to 5.1% in the year 2015 (*P* < .001); and participants who received HIV counseling, testing, and condom in past 12 months decreased from 87.5% in the year 2013 to 78.9% in the year 2014, and rose to 92.2% in the year 2015 (*P* < .001). Prevalence of syphilis increased from 9.3% in the year 2013 to 9.8% in the year 2014, and dropped to 6.1% in the year 2015 (*P* =  .001), and HIV prevalence rose consistently from 6.6% in the year 2013 to 11.2% in the year 2015 (*P* <  .001).

**Table 2:**
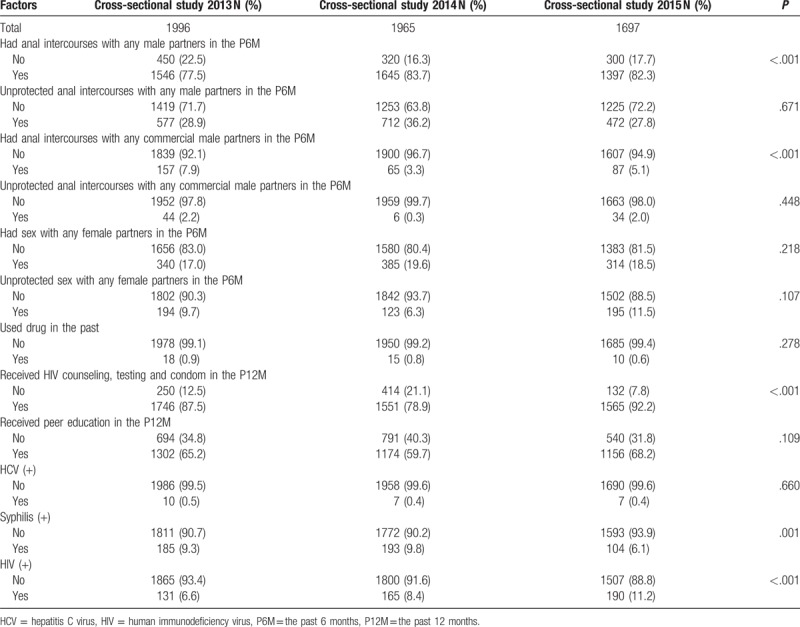
Behavioral characteristics and HIV and Syphilis status of participants in cross-sectional study 2013, 2014 and 2015.

Table 3 presents the Univariate and Multivariate logistic analyses of variables associated with HIV infection among MSM in southwest China. Multivariate logistic regression analyses indicated that compared with HIV-negative MSM, HIV-infected MSM were significantly, more likely, to perform unprotected anal intercourses with any commercial male partners in the past 6 months (adjusted odds ratio (AOR) = 1.81, 95% CI: 1.50–2.20), had sex with any female partners in the past 6 months (AOR = 1.31, 95% CI: 1.01–1.71), used drugs in the past (AOR = 2.73, 95% CI: 1.30–5.71), and are syphilis infected (AOR = 3.53, 95% CI: 2.77–4.49).

**Table 3:**
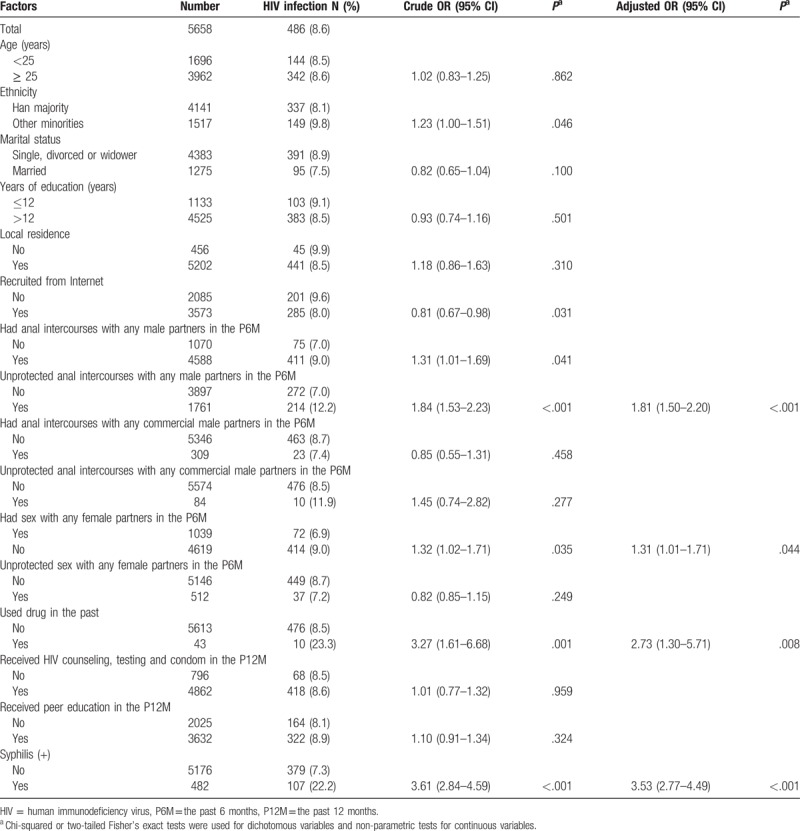
HIV infection and associated factors among study participants in cross-sectional study 2013, 2014 and 2015.

## 4 Discussion

Since HIV started as a drug-driven epidemic, and shifted to mainly, sexual transmission,^[26,27]^ our study focuses on the sexual transmission route by the MSM group. In this cross-sectional study, the data and analysis first indicate that MSM are at higher risk of acquiring HIV, and syphilis infections in Guangxi. Between the years 2013 to 2015, the percent of acquiring HIV infection increased alarmingly, in Guangxi. It rose from 6.6% in the year 2013 to 11.2% in the year 2015. The rise in HIV infections could be due to increase in high-risk behaviors, and wider use of the internet to find partners.^[28,29]^ When compared with previous studies, syphilis infection rates are much lower than HIV infection rates among MSM in China.^[30,31]^ The syphilis infection rose slightly, between the years 2013 to 2014 but dropped down by more than 3% in the year 2015. As seen in previous studies in China, syphilis infection seems to drop down with passing years.^[32,33]^ This could be attributed to the implementation of the syphilis prevention and control plan by the China's Ministry of Health.^[34]^ The strategies included are early screening and appropriate treatment among high risk-populations to prevent the transmission of syphilis.^[28,35]^ Implementation of these strategies might have minimized the occurrence of syphilis among MSM. A study by Chen et al has suggested that preventive strategies towards syphilis will help to control HIV, as individuals with syphilis have the greater risk of acquiring HIV.^32^ Furthermore, a study conducted among MSM in Shenzhen showed that prevalence of HIV, and syphilis infections among MSM with a single partner in the last 6 months is less than those with multiple partners.^[36]^ HCV decreased slightly between the years 2013 to 2014, and remained constant in the year 2015. However, previous studies conducted on MSM population in Beijing indicated high-rise in HCV. It increased from 0.4% in 2004 to 5.2% in 2006.^[37]^ The comparative study suggests that HIV is still expanding at a high rate among these cities. Meanwhile, the proportion of reported HIV/AIDS through homosexual intercourse was 6.7% in the year 2012 in Guangxi,^[22]^ and found to be increasing at an alarming rate in our study, which could lead to preventative measures being neglected against HIV pandemic in the future. Thus, HIV among MSM should be given more attention.

Our analysis of socio-demographic, and behavior characteristics corresponding with HIV, and syphilis infections acknowledge several significant associations. MSM who are single, divorced, or widowed have a higher risk of acquiring HIV. This is not consistent with the previous studies, where married MSM have more chances of getting infected with HIV.^[38]^ In this study, the marriage rate is much higher than the national average of 17.0%.^[32,39]^ Also, the percentage of respondents who had sex with any female partner in the past 6 months, and those who had unprotected sex with any female partner in the past 6 months, rose uncertainly, between the years 2013 to 2014. These results are comparatively, low when compared to previous studies conducted in the city of Zhengzhou, where 30.4% respondents said they had unprotected sex with female partners in past 6 months.^[40]^ This proportion of men who have sex with both the sexes serves as a potential connecting link in spreading HIV, and syphilis from high-risk MSM to their female partners, and from there to the general population. Recruitment from the internet is also significant, and constantly, increasing in our study. MSM are more likely, to use the internet to find homosexual partners, which indicates that internet-based intervention programs could be implemented.^[40,41]^ Moreover, social media is helping in obliging to cultural/social norms towards homosexuality, and help reduce the stigma associated with it. And constant advertising HIV awareness, and testing programs on such platforms may encourage HIV awareness, and testing among MSM.^[42]^

High-risk behaviors are common among this population. One risk factor is that in the year 2013, 77.5% had anal intercourse with any male partners in the past 6 months, which rose inconsistently, to 82.3% in the year 2015. The same behavior with commercial male partners decreased mercurially, from 7.9% in the year 2013 to 5.1% in the year 2015. Unprotected anal intercourse with any male partners in the past 6 months also rose from 28.9% to 36.2% between the years 2013 to 2014 and decreased to 27.8% in the year 2015. MSM recruited in the year 2014 have consistently, greater risk behaviors. Participants who received HIV counseling, testing, and condoms in the past 12 months, and those who received peer education in past 12 months increased inconsistently, between the years 2013 to 2015. This could explain why the odds of unprotected anal intercourse with any male partners in the past 6 months increased. This could imply that as the counseling, testing, free condom distribution, and HIV education increases, the percentage of unprotected anal intercourses decreases. This shows a higher need for interventions for MSM such as condom promotion, and distribution, HIV counseling, and AIDS testing. Also, pre-exposure prophylaxis (PrEP) by using an anti retroviral drug like Tenofovir Disoproxyl Fumarate before exposure to the virus is one of the approaches for the prevention of HIV.^[43]^ A study conducted among MSM in Guangxi, 91.9% of participants responded that they would readily, use the PrEP if it is effective, safe, and free of charge.^[44]^ PrEP should be considered for high-risk MSM population. PrEP provides a high level of protection against HIV if combined with condoms and other preventive methods.^[45]^ Although a very expensive, and challenging program,^[46]^ such preventive, and supportive environments can be promoted through government health care programs will help to curb the HIV infection in Guangxi. HIV, and syphilis related information should be readily, and easily, available to MSM in these cities. Drug use in the past decreased by 0.1%, and this variable is found to be insignificant in our study, and proves true that the HIV begin as a drug-driven epidemic, and shifting to mainly, sexual transmission.^[13,26]^

Our study has a number of limitations. Firstly, the standard questionnaire developed in the survey could be objective to some of the participants, as it contained the information regarding their sexual preference, sex partners, the act of sex itself, and the use of drugs. This could create objective bias. Secondly, the questions that depend on retrospective self-reports like those incorporating activities within past 6 months, and past 12 months could be subjected to recall bias. Also, personal, and sensitive information like sexual activities, number of partners, etc., are susceptible to non-reporting, or under-reporting.

## 5 Conclusion

The 3 annual cross-sectional surveys showed rapidly, spreading HIV among MSM in Guangxi. There is a great need of urgency to introduce more preventative measures against HIV among MSM in the future. These preventive interventions should educate this population about HIV, behavioral risks, and safe sex. Our study shows that intervention strategies such as condom distribution, HIV counseling, free MSM friendly HIV testing, and education regarding safe sex, HIV, and other sex-related diseases in Guangxi that will curb, and prevent HIV among MSM. Although Syphilis infection cases showed some fluctuation, and are reducing in number with passing years in our study, strategies should be developed for controlling syphilis, which can increase the chances of acquiring HIV infection.

## Author contributions

**Conceptualization:** Lan Guanghua, Chen Yi, Tang Shuai, Shen Zhiyong, Tang Zhenzhu, Ruan Yuhua, Mohammed A. Yousuf, Fan Wensheng.

**Data curation:** Lan Guanghua, Chen Yi, Tang Shuai, Shen Zhiyong, Tang Zhenzhu, Ruan Yuhua, Mohammed A. Yousuf, Fan Wensheng.

**Formal analysis:** Lan Guanghua, Chen Yi, Tang Shuai, Shen Zhiyong, Tang Zhenzhu, Ruan Yuhua, Mohammed A. Yousuf, Fan Wensheng.

**Funding acquisition:** Lan Guanghua, Chen Yi, Ruan Yuhua.

**Investigation:** Lan Guanghua, Chen Yi, Tang Shuai, Shen Zhiyong, Tang Zhenzhu, Ruan Yuhua, Mohammed A. Yousuf, Fan Wensheng.

**Methodology:** Lan Guanghua, Chen Yi, Tang Shuai, Shen Zhiyong, Tang Zhenzhu, Ruan Yuhua, Mohammed A. Yousuf, Fan Wensheng.

**Project administration:** Lan Guanghua, Chen Yi, Tang Shuai, Shen Zhiyong, Tang Zhenzhu, Ruan Yuhua, Mohammed A. Yousuf, Fan Wensheng.

**Resources:** Lan Guanghua, Chen Yi, Tang Shuai, Shen Zhiyong, Tang Zhenzhu, Ruan Yuhua, Fan Wensheng.

**Software:** Lan Guanghua, Chen Yi, Tang Shuai, Shen Zhiyong, Tang Zhenzhu, Ruan Yuhua, Mohammed A. Yousuf, Fan Wensheng.

**Supervision:** Lan Guanghua, Chen Yi, Tang Shuai, Shen Zhiyong, Tang Zhenzhu, Ruan Yuhua, Mohammed A. Yousuf, Fan Wensheng.

**Validation:** Lan Guanghua, Chen Yi, Tang Shuai, Shen Zhiyong, Tang Zhenzhu, Ruan Yuhua, Mohammed A, Yousuf, Fan Wensheng.

**Visualization:** Lan Guanghua, Chen Yi, Tang Shuai, Shen Zhiyong, Tang Zhenzhu, Ruan Yuhua, Mohammed A. Yousuf, Fan Wensheng.

**Writing**–**original draft:** Ruan Yuhua, Mohammed A. Yousuf, Fan Wensheng.

**Writing**–**review & editing:** Lan Guanghua, Chen Yi, Tang Shuai, Shen Zhiyong, Tang Zhenzhu, Ruan Yuhua, Mohammed A. Yousuf, Fan Wensheng.

**Contributorship Statement:**

Guanghua Lan, Yi Chen, Zhiyong Shen and Zhenzhu Tang were responsible for conception and design of this study.

Yi Chen, Zhiyong Shen, Zhenzhu Tang, Guanghua Lan, Shuai Tang performed the study.

Yi Chen, Zhiyong Shen, Zhenzhu Tang, Yuhua Ruan, Yousuf Mohammed Adnan and Wensheng Fan involved in the data cleaning and statistical analysis.

Guanghua Lan, Yi Chen, Zhiyong Shen, Zhenzhu Tang, Yuhua Ruan, Yousuf Mohammed Adnan and Wensheng Fan accomplished the drafted manuscript.

All the authors have reviewed and approved the final manuscript.
